# Effect of Sisal Fiber and Polyurethane Admixture on the Strength and Mechanical Behavior of Sand

**DOI:** 10.3390/polym10101121

**Published:** 2018-10-10

**Authors:** Jihong Wei, Fanxuan Kong, Jin Liu, Zhihao Chen, Debi Prasanna Kanungo, Xiaowei Lan, Canhui Jiang, Xiao Shi

**Affiliations:** 1 School of Earth Sciences and Engineering, Hohai University, Nanjing 210098, China; weijhhhu@163.com (J.W.); kfxhhu@163.com (F.K.); hhuczh@163.com (Z.C.); wcakpk56@163.com (X.L.); Jcanhhhu@163.com (C.J.); hhushix@163.com (X.S.); 2 CSIR-Central Building Research Institute (CBRI), Roorkee 247667, India; debi.kanungo@gmail.com

**Keywords:** fiber reinforcement, polymer treatment, unconfined compressive strength, interfacial interaction, mechanical behavior

## Abstract

One major problem related to sandy soil is its low shear strength and cohesion in engineering. Although much effort has been made to strengthen sand mass with satisfactory performances, most undertakings lack environmental considerations. Thus, a combination of natural fiber and macromolecule polymer material attempts to achieve both strength and eco-friendliness. In the present investigation, sisal fiber (SF) and water-based polyurethane (PU) were used to reinforce sand. A series of unconfined compression tests were carried out on sand specimens at different percentages of fiber contents (0.2%, 0.4%, 0.6%, and 0.8% by weight of dry sand) and polymer contents (1%, 2%, 3%, and 4% by weight of dry sand). The results showed within our test range that the unconfined compressive strength (UCS) as well as post-peak strength of specimens increase with fiber and polymer contents. The inclusion of fiber and polymer significantly improve the ductility of specimens. The effect of dry densities on UCS were studied with three proportions. It is found that a high dry density led to an increase of UCS due to an effective contact area increase. The interactions were studied by observation through scanning electron microscopy (SEM) images. The presence of water-based polyurethane has the potential to improve the interparticle cohesion of sand due to its unique network membrane structure. The fiber reinforcement benefit depends strongly on the friction, interlocking force, and bond strength at the interface.

## 1. Introduction

Soil can often be regarded as a combination of four basic types: gravel, sand, clay, and silt [1]. It generally has poor mechanical properties (e.g., shear and compressive strength), and its characteristics may be affected strongly by its surroundings. Therefore, from the perspective of geotechnical engineering, soil reinforcement is essential for improving the engineering characteristics of soil such as shear strength, compressibility, hydraulic conductivity, etc. [2], and so as to meet the requirements of engineering. Especially for sandy soil, it is characterized by a poorly graded, loose structure and high hydraulic conductivity, which exacerbates its instability, leading to more susceptibility to environmental impacts and thereby causing geological and/or engineering disasters (e.g., liquefaction, differential settlement, and erosion). Thus, it is very important to improve the stability of sand mass by reinforcement.

It is known that the clay content of sandy soil is relatively low, while sand content is higher than 50%, resulting in low interparticle cohesion. Generally, the structural binding and aggregation between soil particles are mainly conditioned by the higher amounts of clay particles and organic content in soils [3]. Thus, improving soil strength by enhancing its cohesion can be considered as a good countermeasure. The conventional approach is to adopt chemical stabilizers, including lime, cement, and fly ash, which are proven to improve the strength of soil [4,5,6,7]. However, these chemicals may lead to soils showing high stiffness and brittleness [8,9], as well as negative impacts on the natural environment. As an alternative, polymeric materials are introduced to enhance sand cohesion and can form a unitary coherent matrix with much smaller environmental impacts (e.g., water pollution and vegetation growth) than the above additives. Previous studies have shown the possibility of using polymers to strengthen soil, reduce permeability, and control soil erosion [10,11,12,13,14,15]. This is mostly benefited from the particle elements being closely joined and finely structured by these polymeric matters.

On the other hand, it has a long-applied history for including randomly distributed, discrete fibers into the matrix (i.e., soil mass) to provide an improvement in the mechanical behavior of the soil composite. Many research studies have received encouraging results that prove the effective usage of fibers for soil reinforcement [16,17,18,19]. So, both natural and synthetic fibers are widely used and attract increasing attention in geotechnical practices due to their convenient usage (i.e., simple mixing procedure), cost saving, and good mechanical properties. One of the main advantages of using randomly distributed fibers is the maintenance of strength isotropy and the absence of potential planes of weakness that can develop parallel to the oriented reinforcement [20]. However, the strategy in using synthetic fiber (e.g., polypropylene fiber, which is most commonly used) for soil reinforcement, may lead to environmental problems (e.g., microplastic pollution) if there are no reasonable and effective controls [21]. By comparison, the natural fibers such as coconut, sisal, palm, etc., not only impart great strength to the soils, they also achieve eco-friendliness. Beyond these stated advantages, there are still some drawbacks of natural fibers in the practical application, such as their biodegradability. However, this problem can be overcome by chemically treating the fibers.

Though a good performance of soils with fiber and chemical additives (i.e., cement, lime, and fly ash) has been observed in many research studies, it is expected that the combination of fiber and polymer can achieve better enhancement effect on the strength of sand through their synergetic effect. In this attempt, a sisal fiber–polyurethane polymer mixture is a further step in this direction.

In this paper, a series of laboratory studies were performed to further understand the effects of randomly distributed sisal fiber (SF) and polyurethane (PU) for improving the unconfined compressive strength of sand. The unconfined compression tests were carried out on sand specimens with different amounts of fibers and polymers. The scanning electron microscopy (SEM) tests were also conducted to reveal the internal structure of reinforced sand mass and primarily investigate its strengthening mechanism.

## 2. Materials and Methods

### 2.1. Materials

The sand used in the present experiment was taken from the area of Nanjing, China. It was oven-dried, crushed, and passed through a two-mm sieve in the laboratory for spare. The physical properties of sand are listed in Table 1.

The polymer used in the experimental tests was water-based polyurethane. Starting from polyethylene glycol (PEG, Jining Hongming Chemical Reagent Co., Ltd., Jining, China), polypropylene glycol (PPG, Shanghai Ika Biotechnology Co., Ltd., Shanghai, China), and toluene diisocyanate (TDI, Nantong Runfeng Petrochemical Co., Ltd., Nantong, China), the prepolymer was synthesized via condensation polymerization. Due to the hydrophilic segments or groups in the molecular chain, the prepolymer can better respond to water. Thus, stable polyurea was prepared via emulsification in water and chain growth. The physical properties of the polymer are summarized in Table 2.

**Figure 1:**
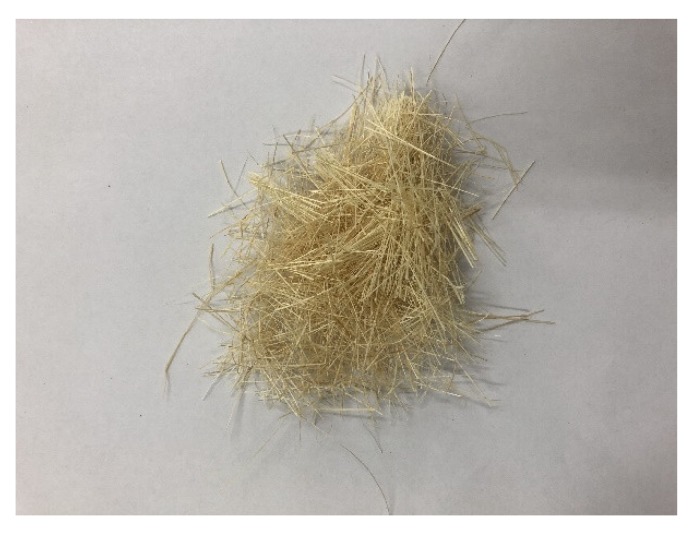
Photograph of the short sisal fiber.

The photograph of the short SF (Guangxi Qianglimaye Co., Ltd., Chongzuo, China) is shown in Figure 1. The physical and mechanical properties of the SF are listed in Table 3 [1]. A fiber length of 18 mm was adopted for the preliminary study.

### 2.2. Preparation of Specimens

For evaluating the effect of different SF and PU content on strength behavior, the orthogonal test was carried out with four SF contents ($p_{f}$ = 0.2%, 0.4%, 0.6%, and 0.8% by weight of dry sand) and PU contents ($p_{p}$ = 1%, 2%, 3%, and 4% by weight of dry sand). All of the test specimens were compacted at a dry density of 1.5 g/cm^3^ and an initial water content of 10% for easy demolding. With respect to the effect of dry density, four different values adopted for $\rho_{d}$ were 1.45 g/cm^3^, 1.5 g/cm^3^, 1.55 g/cm^3^, and 1.6 g/cm^3^. To control the number of test specimens, three specific proportions of SF and PU were adopted with $p_{f}$ and $p_{p}$ such as 0.4–2%, 0.6–2%, and 0.4–4%, respectively. The other conditions (i.e., initial water content) remained unchanged. Thus, a total of 25 groups of sand specimens with SF and PU at different percentages were made for performing the unconfined compression tests.

If only fibers were admixed, the sand specimens could be formed via a compacting and capillary effect, but with the action of external force, they still perform extremely destructive characteristics. Therefore, only polymers and fiber mixed-sand specimens were prepared for the experiments. The sand mixture was prepared by hand mixing the required amount of dried natural sand, distilled water, polymers, and fibers. Sisal fibers were first mixed with dry sand thoroughly. Then, an additional polymer emulsion was added to form a uniform soil structure, and homogeneous mixtures were obtained. All of the mixtures were placed in the mold and compacted prior to the complete solidification of polymers. After the compaction, the specimens treated with PU were air dried at room temperature (i.e., 25 ± 1 ℃) without exposure to sunlight for 48 h until tests. By checking the weight change of specimens over time, it was found that the water content of all of the specimens gradually converged to a certain range (i.e., below 2%).

### 2.3. Testing Program

#### 2.3.1. Unconfined Compression Tests

The common unconfined compression apparatus was employed in the tests. In order to ensure the specimens were compacted uniformly, the required amounts of sand mixtures were placed inside the mold, static compression was done in three steps based on American Society for Testing Material (ASTM) standards (D2166-00), and shaped into a cylinder (i.e., diameter 39.1 mm × height 80 mm). The unconfined compression tests were conducted at a constant loading rate (2.4 mm/min) until the specimen achieved an axial strain of 20% (i.e., vertical deformation of 1.6 cm). During this process, an axial displacement meter with a capacity of 10 mm and an accuracy of 0.01 mm was placed to record the axial deformation of specimen; thus, the axial strain (ε) of the specimen can be calculated by the following:

(1) $$\varepsilon =\frac{\Delta h}{h_{0}}\times 100\%$$

where Δh (mm) = length change of specimen as read from deformation indicator, and $h_{0}$ (mm) = an initial length of the test specimen. Additionally, three specimens with identical SF and PU content were involved in each test group for reducing the accidental error, and the average value of these three individual strengths was accepted as a result.

#### 2.3.2. Scanning Electron Microscopy (SEM) Tests

Both specimens with 0.6% SF-4% PU content and without fiber addition were sampled into 1 cm^3^ (i.e., length 10 mm × width 10 mm × height 10 mm) cubes after performing the unconfined compression test. Prior to the examination, the samples were dehydrated and treated with gold coating to enhance the electrical conductivity. Microscopic images of the PU and SF-reinforced sand were recorded to observe its internal microstructure and fiber reinforcement surface; thus, the synergistic effect of fiber and polymer on the strength and compressive deformation behavior of the specimen could be investigated.

## 3. Results and Discussions

### 3.1. Unconfined Compression Test Results

As mentioned above, 25 groups of unconfined compression tests were conducted to investigate the effect of fiber, polymer, and dry density on the strength and mechanical behavior of reinforced sand, and the corresponding test results are summarized in Table 4 and Table 5. Note that the axial stress corresponding to 15% axial strain is considered the unconfined compressive strength if there exists no or less apparent peak axial stress on the stress–strain curve. By the integral method, the compressive energy of specimens with different SF and PU content in the whole compressive process (strain to 20%) was calculated and presented in Table 4, more clearly showing the compressive performance of specimen.

**Figure 2:**
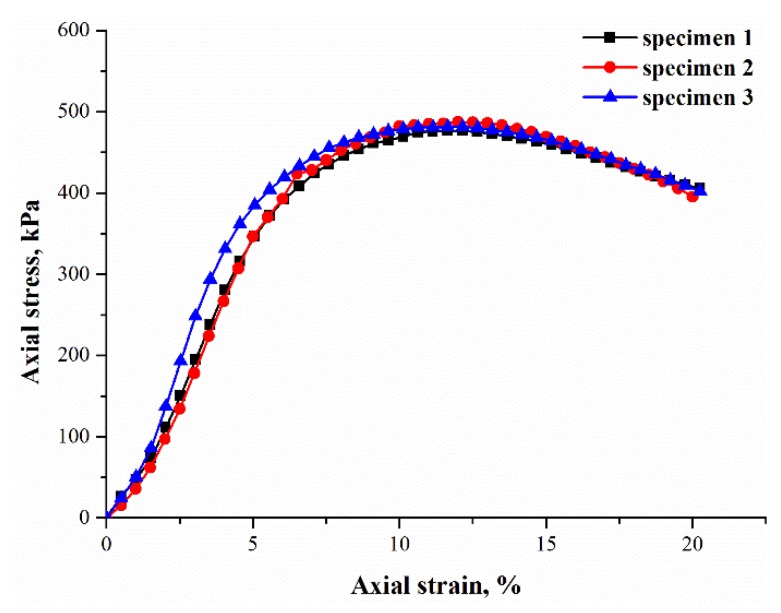
Stress–strain curves of three parallel specimens: ($p_{f}$ = 0.2%, $p_{p}$ = 3%, and $\rho_{d}$ = 1.5 g/cm^3^).

To ensure the repeatability of the tests, the stress–strain curves of the three parallel specimens prepared at the same conditions (i.e., $p_{f}$ = 0.2%, $p_{p}$ = 3%, $\rho_{d}$ = 1.5 g/cm^3^, and 10% initial water content) were selected for comparison, as given in Figure 2. It can be observed that the stress–strain curves of the parallel specimens have the same varying trends with little differences amongst them (below 10%), even though the fibers were randomly oriented, demonstrating an identical rule in the test process. Moreover, the other groups of specimens showed this repeatability as well. Therefore, there is only one typical curve representing each group, as illustrated in the following sections.

### 3.2. Effect of Fiber Content on the Unconfined Compressive Strength of Sand

**Figure 3:**
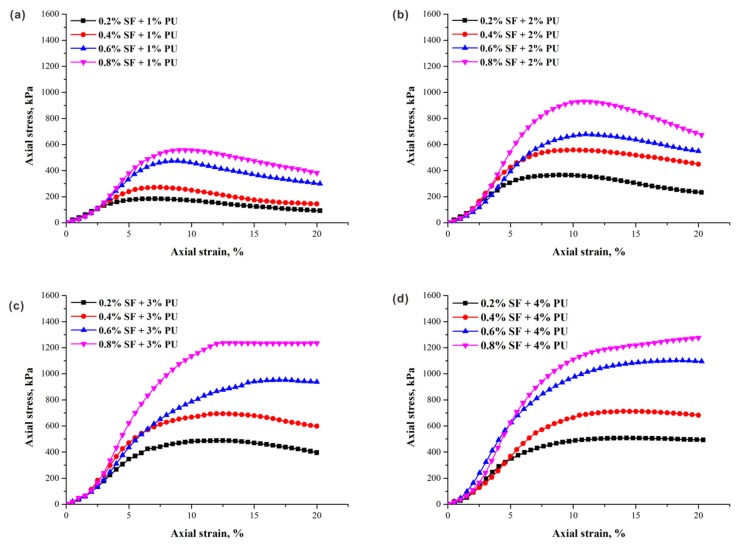
Typical stress–strain curves of specimens with varying sisal fiber content and (**a**) 1% PU content; (**b**) 2% PU content; (**c**) 3% PU content; and (**d**) 4% PU content at 1.5 g/cm^3^ dry density.

The typical stress–strain curves of specimens ($\rho_{d}$ = 1.5 g/cm^3^) with different fiber contents obtained from unconfined compression tests are presented in Figure 3. The contributions of fiber increment to peak axial stress is significant under the same conditions. As illustrated, regardless of PU content, the peak axial stresses increase as the fiber content increases. For specimens with a lower PU content, the axial stress increased monotonically with the increase of axial strain until the unconfined compressive strength (peak axial stress) was reached, and then decreased (Figure 3a,b). The stress–strain curves of these specimens under axial loading behaved with strain softening characteristics, showing brittle behavior. However, a completely different trend is observed under the condition of higher PU content, as the axial stress increased with axial strain and gradually slowed down, demonstrating the strain-hardening characteristics (Figure 3c,d). This difference is contributed by two factors: the better interconnection between fibers and the sand matrix with the help of the PU, and the stronger interfacial forces produced by the more effective contact area with the increase in fiber content. As a result, an increase in the failure strain corresponding to the strength can be obviously observed. For example, the failure strain varied from 8.8% to 10.9% when the fiber content increased from 0.2% to 0.8% in Figure 3b. Additionally, the residual strength also performed an increasing trend with increasing fiber content, which is attributed to the improvement of the fiber reinforcement in the material ductility.

An increase in fiber content leads to an increase in the compressive energy of the specimen, indicating the good toughness of specimens with higher fiber content, which require more energy to deform. In the presence of fiber, the radial deformation of the specimen during compression was effectively controlled. Although more fibers provided much stronger reinforcing effect, the slope of the curves before 4% axial strain were not significantly affected by the increase in fiber content. A previous study observed this since the fibers in the soil matrix have low elastic stiffness, which provides stronger ability in energy absorption when the external force acts; thus, the frictional resistance is not realized because of the insufficient relative displacement between the fibers and soil matrices [22]. Therefore, a greater stiffness of fiber is required for contributing to the earlier anti-destruction capacity of the specimens.

**Figure 4:**
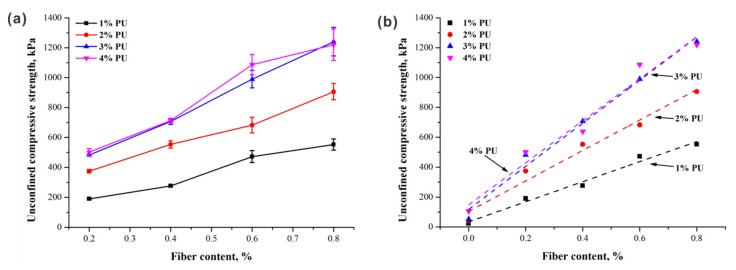
Test results of (**a**) effect of fiber content on unconfined compressive strength (UCS) of PU treated specimens at 1.5 g/cm^3^ dry density; (**b**) linear fitting for the relationship between the UCS and fiber content.

The critical issue affecting sand strength is the loss of clay particles (i.e., a decrease of the interparticle cohesion of the soil); thus, it is important to meet the requirements of improving the mechanical properties of sand by enhancing the cohesion to a certain measure [23]. Undoubtedly, the cohesion provided by PU is the dominant factor controlling the fiber reinforcement benefit. Variations in unconfined compressive strength (UCS) with fiber content for specimens with different PU contents are drawn in Figure 4a. It can be observed that the UCS almost linearly increased with the increase of fiber content, regardless of PU content. For better comparison, the UCS of PU-treated sand without fiber inclusion, taken from Liu et al. [24], is summarized in Table 6. A good linear relationship between the UCS and fiber content was found, as shown in Figure 4b. Therefore, the UCS of fiber–polymer-reinforced sand can be considered to be composed of two parts: the UCS of polymer-treated sand and the UCS increment induced by the addition of fiber. Moreover, the increase rate (i.e., the slope of the fitting curve) also had a slight increase with the increase in PU content; however, this change became relatively weak beyond 3% PU content, suggesting that there is an upper limit for the beneficial effect of polymer treatment. The effect of PU content will be discussed in the following section.

**Figure 5:**
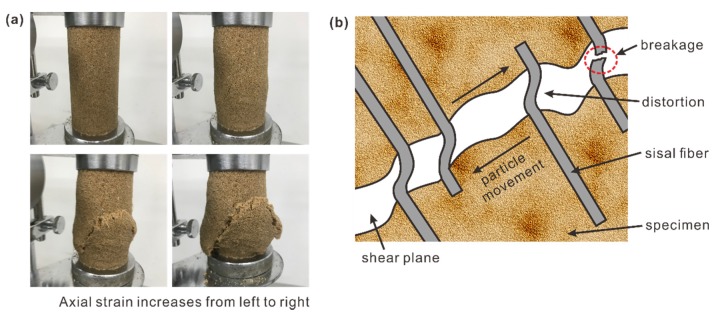
Results of fiber–polymer-reinforced sand in the unconfined compression tests. (**a**) Photographs of specimens with the increase of axial strain during tests ($p_{f}$ = 0.8%, $p_{p}$ = 1%, and $\rho_{d}$ = 1.5 g/cm^3^); (**b**) Schematic drawing of fiber reinforcement in the test process.

With respect to the effect of fiber content on failure characteristics, the failure process and mode of specimens subjected to axial stress are revealed in Figure 5 to primarily analyze its damage mechanism. As shown in Figure 5a, the failure cracks triggered by the increasing axial stress gradually developed due to the interfacial shear stress. These cracks became wide and long, which indicated that shear failure was translated into a tensile one, and finally broke through the whole specimen. The beneficial effect of fibers in this process can be regarded as bridges to efficiently retard the formation of shear surface in the sand matrix and impede the further opening and development of cracks through the individual strength of embedded fibers [25]. For better understanding of the fiber reinforcement benefit during the tests, a schematic drawing is presented in Figure 5b. For simplicity and clarity, it is assumed that the fiber is perpendicular to the failure plane. As illustrated, due to the rotation and rearrangement of sand particles under the axial stress, shear stress is first applied, making fibers that are located near the shear plane bend, deform, or even break out, which also drives a pull-out force on the embedded fibers. With the development of this crack (i.e., through the crack), the fibers through the opening are subjected to elastic extension induced by the tensile force. It is believed that the effectiveness of fiber reinforcement in unconfined compression tests can be improved as a result of the interfacial mechanical interactions between the fibers, the sand matrix, and the tensile strength of the fiber. Therefore, the pull-out resistance conditioned by the bond strength, friction, and interlocking force between fibers and the sand matrix is partially responsible for the improvement in compressional behavior, especially the post-peak strength. It can explain why the residual strength has an obvious increase with increasing fiber content. Fiber inclusion decreases stiffness and changes the brittle behavior to a more ductile one, thereby improving the stress tolerance and compressional behavior of the specimen, which is consistent with many other research studies [26,27,28].

In general, the shear cracks randomly intersect the fiber, leading to extremely variable anchoring lengths. Along the shear plane, the debonding and pull-out failure of fibers are prone to take place on one end, which has a shorter effective anchorage length on the side of the failure plane. Due to the low skin frictional resistance of the fiber having the shortest embedded lengths, the fiber slippage in composites becomes increasingly susceptible to being caused by the mobilized tensile stress developed in the fiber. Such pull-out failure makes fibers unable to give full play to the reinforcement effect. As a matter of fact, the real situation of randomly distributed fibers in soil is more complicated than the employed assumption due to the variation of the intersection angle between the fiber and the failure plane. More extensive studies should be conducted to refine the results.

Accordingly, it seems that fiber length is also a non-negligible parameter affecting the compressional behavior of the specimen [29,30]. Previous study showed that beyond 20 mm, a further increase in fiber length may cause a reduction in the deviator stress by reducing the soil–fiber interlocking; thus, there is an optimum value for fiber length [31]. It is also expected that excessive fiber length also leads to the tangling of fibers, which reduces the mixing of the soil and thus makes it more difficult to obtain a homogenous mixture. Further studies are needed to evaluate the effect of fiber length.

### 3.3. Effect of Polymer Content on the Unconfined Compressive Strength of Sand

**Figure 6:**
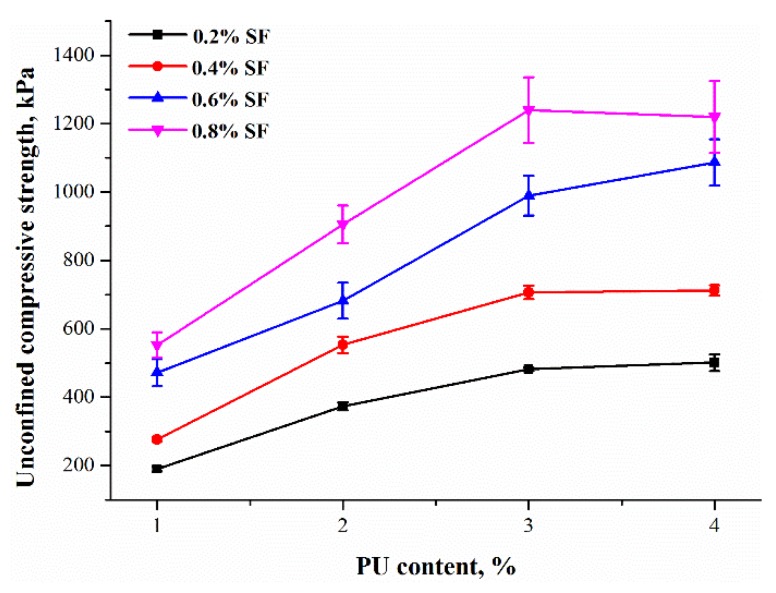
The relationship between the UCS and PU content.

The corresponding UCS is presented in Figure 6 as PU content increases. It indicates that polymers play an important role in strength enhancement. For example, as PU content increases from 1% to 4%, the values of UCS for specimens with 0.2% and 0.8% fiber content increase significantly from 190.07 kPa to 501.33 kPa and from 552.33 kPa to 1219.86 kPa, respectively. It seems that PU treatment has possibilities of providing a satisfactory (i.e., promoted) structural environment for fiber reinforcement. A decrease or slight increase in strength growth were observed with higher PU content (3% and 4%, respectively) due to the limited interval spaces between loosened sand particles at a fixed dry density.

**Figure 7:**
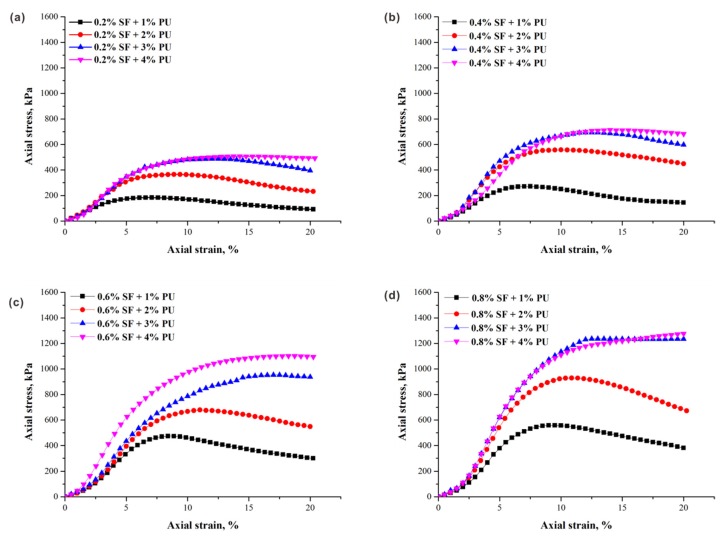
Typical stress–strain curves of specimens with varying PU content: (**a**) 0.2% fiber content; (**b**) 0.4% fiber content; (**c**) 0.6% fiber content; and (**d**) 0.8% fiber content at 1.5 g/cm^3^ dry density.

Figure 7 shows the typical stress–strain curves of specimens ($\rho_{d}$ = 1.5 g/cm^3^) treated with different PU contents at the same fiber contents. It can be observed that an increase in the PU content leads to a great enhancement in peak axial stress. However, as mentioned above, at a certain point (i.e., beyond 3% PU content), the strength reaches a plateau and the subsequent gains become much harder to get because of complete pore filling, which restricts the improvement efficiency of PU. It should be noted that the PU content has a great influence on the overall shape of the stress–strain curves. By comparison, strain hardening is more obvious with the increase of the PU content, suggesting a high toughness and strain resistance for treated sand. Obviously, PU treatment has a stronger effect on reforming the internal structure of the sand body compared with fiber reinforcement. This is because the cohesion is affected slightly by addition of fiber, being basically a function of cementation induced by polymers [32]. Greater amounts of polymeric material in sand possess a stronger capacity to resist deformation by enhancing structural binding and aggregation between sand particles, resulting in a dense particle arrangement with stronger constrains. The reduction in the loss of post-peak stress is more pronounced for lower PU content. This is because the interfacial bonding strength between fiber and soil is not strong enough to prevent fiber slippage.

**Figure 8:**
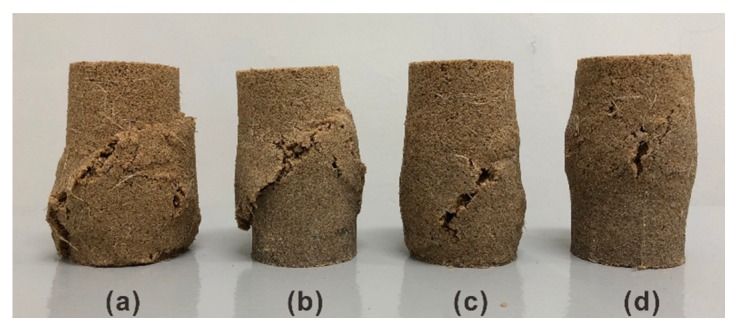
Variation of the failure characteristics of specimens treated with different PU content. (**a**) 0.6% fiber–1% polymer; (**b**) 0.6% fiber–2% polymer; (**c**) 0.6% fiber–3% polymer; and (**d**) 0.6% fiber–4% polymer.

Due to the presence of polymers, the sand particles are finely structured; thus, they exhibit a better strength behavior and show sufficient ductility. As shown in Figure 8, the difference in the size of the cracks in the surface of four specimens after compressional failure is observed. The specimen with relatively low PU content (i.e., 1% and 2%) has a main crack that comes through the surface of the specimen (Figure 8a,b). In contrast, these big cracks dwindle with increasing PU content, indicating less disturbances and particle movements along the failure plane during the compression process (Figure 8c,d). Similar behavior was observed for other reinforced specimens. As a result, the specimens are maintained as mostly intact, and are still powerful enough to provide certain strain resistance, which is in good agreement with the corresponding stress–strain curves.

The reduction in brittleness can be attributed to the interparticle desiccation induced by the elastic polymeric materials [33]. After solidification and formation, an elastic connection is formed between sand particles, which prevents the specimens from being damaged due to relatively great local stress by distributing the stress to a broader area through a more interfacial effective contact area. The detachment and movement of particles from the matrix can be effectively prevented through the strong bonds provided by the PU between sand particles, resulting in high resistance against external forces. Therefore, these bonds are regarded as displaying a buffer with absorbing and moderating impacts (i.e., the effective stress transmitted through sand particles) via elastic deformation. It is expected that PU treatment is more useful in impeding the development of microcracks, which is limited by these bonds between particles only withstanding relatively small stretch distortion.

### 3.4. Effect of Dry Density on the Unconfined Compressive Strength of Sand

**Figure 9:**
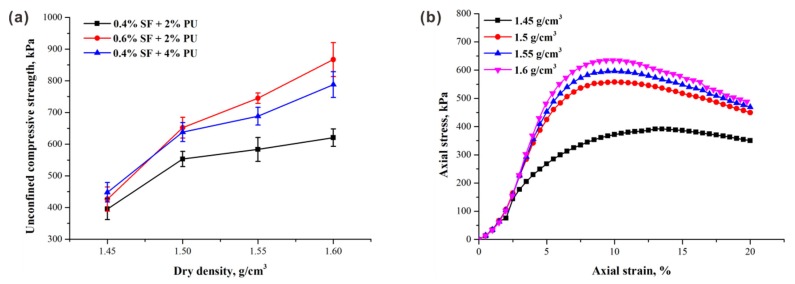
Results of unconfined compression tests in fiber–polymer-reinforced sand: (**a**) relationship between UCS and sand dry density; (**b**) Typical stress–strain curves of specimens at different dry densities ($p_{f}$ = 0.4%, $p_{p}$ = 2%).

Figure 9 shows the results of unconfined compression tests of specimens tested at different dry densities. It can be seen from Figure 9a that the UCS of specimens increases with increasing dry densities. The specimen with relatively high fiber content has a better performance, indicating the fiber’s good response to dry density. Moreover, although dry density does not significantly affect the overall shape of the stress–strain curves, an increase in the peak stress and residual strength can be observed. Similar results were also found in other groups.

The UCS increases with dry density can be attributed to the contact conditions. The required compaction is determined by dry density, which means that greater compaction is needed for the specimens with the same dimension to achieve higher dry density. This leads to a lower void ratio and a smaller pore diameter; thus, it gives rise to the effective contact area between the sand particles. The efficiency of both fibers and polymers is achieved by the contact conditions of sand particles. A previous study showed that the effective contact area directly influences the magnitude of the interfacial friction and adhesion [34]. For fiber reinforcement, the interfacial mechanical interaction and interfacial shear resistance on the fiber–sand interface are improved as a result of the higher contact force and interlocking between the adjacent sand particles [35]. On the other hand, due to relatively low porosity, stronger interparticle stresses between sand particles are created via the adhesion of polymers. This is consistent with the observation of Liu et al. [36] that both the cohesion and compressive strength of polymer-treated sand specimens are significantly improved with increasing dry density. Therefore, higher dry density performs its role in further enhancing the UCS of fiber–polymer-reinforced sand in a positive way.

### 3.5. Combined Mechanism of Fiber–Polymer-Reinforced Sand

**Figure 10:**
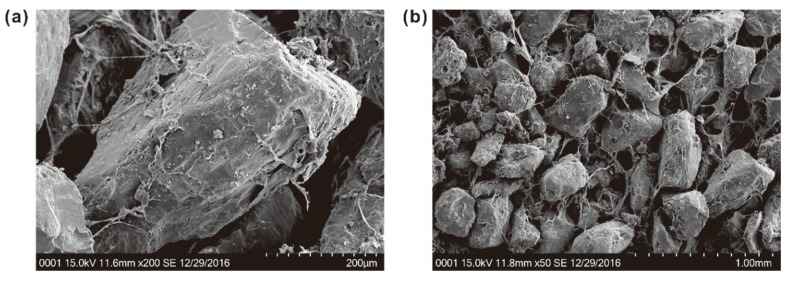
SEM images of sand treated with PU: (**a**) interaction characteristics between polymers and sand particles; (**b**) Internal microstructure of treated sand.

Generally, the organic matter in soil affects the soil structure [37]. Figure 10 shows the microstructure of sand specimens treated with PU. It can be observed clearly that there is a thin layer of membrane coating closely interfaced on the surface of sand particle so that it is not liable to detach from sand particles (Figure 10a). Furthermore, the pores between particles are filled with these membranes, which leads to adjacent sand particles clustering and forming a whole system. It is believed that this membrane is a lightweight flexible material that acts as an effective energy transfer path, providing adequate retention and proper stress distribution. Later, by taking the sand particles as the base point and the membrane as interconnecting strands, a complex framework is constructed from polymeric materials, and then formed into honeycomb and space network structures, performing a good load-bearing role (Figure 10b). As a result, the particles are held so firmly due to a series of mutual criss-crossing and interconnected membranes that their motion is restricted at fixed positions. It is expected that these membranes may become stronger and stiffer during the drying process, thus promoting the interparticle stresses between sand particles. This leads to the strength of the specimen increasing over time, which agrees with the observation of Liu et al. [38].

The presence of polymers has ability to enhance sand strength with the provision of sufficient cohesive force, a dense arrangement of sand particles, and a good impact absorption capacity due to its unique structure. Therefore, the interaction between sand particles and polymers is mainly conditioned by the following factors: (1) particle micromorphology (e.g., the surface characteristic) and grain size distribution, which directly or indirectly affect the adhesion effect (i.e., bonding effect) of polymers on sand particles; (2) ambient temperature, which may accelerate the fragmentation and detachment of membranes from sand particles under high temperature (i.e., exceeds 50 ℃); (3) the structural flexibility of the polymers, which allows intermolecular forces to take place with higher effectiveness [23]; (4) the number of hydrophilic groups (e.g., hydroxyl) on macromolecular chains, which affects the adsorption capacity of free water molecules through hydrogen bonding, and thus weakens the interfacial adhesive force of membranes through lubrication.

**Figure 11:**
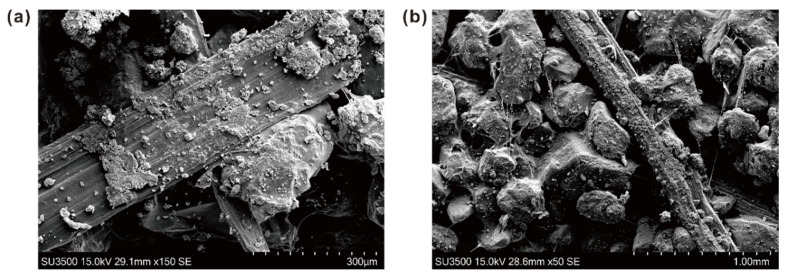
SEM images of fiber–polymer-reinforced sand ($p_{f}$ = 0.6%, $p_{p}$ = 4%, and $\rho_{d}$ = 1.5 g/cm^3^). (**a**) Fiber surface in polymer-treated sand; (**b**) Interaction characteristics between fibers and sand particles.

SEM images of fiber–polymer-reinforced sand are presented in Figure 11. As shown in Figure 11a, some parallel shallow grooves distributed along the fiber axis are observed on the fiber surface. It is known that the interfacial roughness plays an important role in the interfacial mechanical behavior [39,40]. Thus, the rough fiber surface provides high resistance to interfacial shear stress by enhancing the friction on the fiber–matrix interface, resulting in the increased strength of the specimen. Besides, the increased roughness of the fiber interface constructs a mechanical interlock and resists the relative movement of fibers in the composites [41], which is exactly the surface characteristic of sisal fiber. Moreover, it can also be observed that the fiber surface is also wrapped by membranes (Figure 11a). As mentioned above, the effective area between the fiber and the matrix is also a key factor affecting the interfacial friction and interlock. Owing to the presence of these membranes, fibers are bonded tightly with sand particles, causing a more effective contact area and thereby increasing the interfacial friction and interlocking force (Figure 11b). Compared with natural sand, fibers in this environment are more prone to bear stress through an anchorage effect instead of producing relative slippage in the matrix. Overall, friction, interlock force, and bond strength at the interface are the dominant factors controlling the fiber reinforcement benefit.

Within our test range, contrary to the variation of UCS with PU content, the UCS of specimens increases monotonously with fiber content, showing no trend of saturation nor a decreasing trend. However, too many fibers may lead to an adverse effect on the strengthening because the fibers adhere to each other to form lumps, which lead to insufficient contact with particles [31,42]. Further study is needed to determine the critical values.

## 4. Conclusions

A series of unconfined compression tests were conducted to study the unconfined compressive strength and mechanical behavior of short sisal fiber-reinforced and water-based polyurethane-treated sand. The interfacial mechanical interaction between sand particles, fibers, and polymers was investigated. The critical factors affecting polymer treatment and fiber reinforcement were discussed. The main conclusions from the present work can be summarized as follows:

(1) The unconfined compressive strength and residual strength of the sand specimen increase with an increase in the fiber and polymer content within our test range. Owing to the inclusion of sisal fiber and polymer, sand specimens exhibit a better strength behavior and show sufficient ductility.
(2) Water-based polyurethane (PU) treatment is the precondition for fiber reinforcement. It provides a suitable structural environment for fibers by increasing the interparticle cohesion of sand.
(3) An increase in the sand dry density has an affirmative effect on improving the unconfined compressive strength of the specimens. This is mainly attributed to the interfacial interactions between the fiber and the matrix, which is further enhanced due to the more effective contact area at a high, dry density.
(4) The presence of water-based polyurethane (PU) has the potential to improve the interparticle cohesion of sand due to its unique network membrane structure. These elastic polymeric membranes effectively improve the interparticle stress and restrict particle motion through surface enwrapping, pore filling, and a connecting effect.
(5) Fiber reinforcement is conditioned by the interfacial mechanical interactions between the fiber and the matrix. Friction, the interlocking force, and bond strength at the interface are the dominant factors controlling the fiber reinforcement benefit.

Although the interfacial interactions between fibers and polymer–sand composites play an important role in reinforcing soil systems, few research efforts have yet been carried out. A proper understanding of the interaction mechanism along the fiber–soil interfaces can be obtained through quantitively analyzing the interfacial mechanical parameters, such as the interfacial bonding and shear strength. Practical applications are also of great concern, since the laboratory experiments showed this treatment had the potential to improve the mechanical properties of sand by strengthening its internal structure. However, there are many different stimuli in the practical applications, such as temperature, ultraviolet rays, water, chemical mediators, etc. Further research studies are needed to determine whether the polymers are able to adapt to the complex environmental conditions.

## Figures and Tables

**Table 1:** Physical and mechanical properties of sand used in this experiment.

Properties	Values
Specific gravity (g/cm^3^)	2.65
Natural water content (%)	2
Maximum dry density (g/cm^3^)	1.66
Minimum dry density (g/cm^3^)	1.34
Maximum void ratio	0.970
Minimum void ratio	0.590
*Grain size analysis*	
Constrained grain size d_60_	0.3684
Median grain size d_30_	0.2368
Effective grain size d_10_	0.1474
Nonuniformity coefficient C_u_	2.50
Curvature coefficient C_c_	1.03

**Table 2:** Properties of water-based polyurethane (PU).

Properties	Values
Specific gravity (g/cm^3^)	1.18
Viscosity (MPa·s)	650~700
Mass fraction (%)	85
pH	7
Appearance	Light-yellow

**Table 3:** Physical and mechanical properties of the sisal fiber (from Hejazi et al. 2012 [1]).

Properties	Values
Type	Single fiber
Specific gravity (g/cm^3^)	1.2~1.45
Average diameter (mm)	0.25
Average length (mm)	18
Breaking tensile strength (MPa)	560

**Table 4:** Unconfined compressive strength of specimens with 1.5 g/cm^3^ dry density and a 48-h curing time.

Serial Number	$\mathrm{Fiber}\;\mathrm{Content}\;p_{f}\;(\%)$	Unconfined Compressive Strength (kPa)/Compressive Energy (kJ/m^2^)			
		$p_{p}=1\%$	$p_{p}=2\%$	$p_{p}=3\%$	$p_{p}=4\%$
T1-4	0.2	190.07/2.13	373.87/4.43	481.70/6.00	501.33/6.37
T5-8	0.4	276.40/2.97	553.01/6.93	706.90/8.47	712.45/8.44
T9-12	0.6	471.90/5.29	651.89/7.89	989.39/10.50	1086.88/12.97
T13-16	0.8	552.33/6.40	905.79/10.67	1240.24/14.38	1219.86/14.28

**Table 5:** Unconfined compressive strength of specimens with different dry densities and 48-h curing time.

Serial Number	$\mathrm{Fiber}\;\mathrm{Content}\;p_{f}\;(\%)$	$\mathrm{Polymer}\;\mathrm{Content}\;p_{p}\;(\%)$	Unconfined Compressive Strength (kPa)			
			$\rho_{d}=1.45\;g/{\mathrm{cm}}^{3}$	$\rho_{d}=1.5\;g/{\mathrm{cm}}^{3}$	$\rho_{d}=1.55\;g/{\mathrm{cm}}^{3}$	$\rho_{d}=1.6\;g/{\mathrm{cm}}^{3}$
T17-19	0.4	2	395.17	553.01	583.22	620.41
T20-22	0.6	2	426.46	651.89	745.04	866.98
T23-25	0.4	4	447.93	637.86	687.92	787.91

**Table 6:** UCS of PU-treated sand without fiber inclusion (from Liu et al., 2017).

Serial Number	Dry Density (g/cm^3^)	PU Content (%)	UCS (kPa)
S2	1.5	1	22.99
S7	1.5	2	41.88
S12	1.5	3	52.69
S17	1.5	4	106.14
